# New idea for treatment strategies for Barcelona Clinic Liver Cancer stages based on a network meta-analysis

**DOI:** 10.1097/MD.0000000000006950

**Published:** 2017-05-19

**Authors:** Kun Li, Hai-Tao Wang, Yu-Kun He, Tao Guo

**Affiliations:** Department of General Surgery, Zhongnan Hospital of Wuhan University, Wuhan, P.R. China.

**Keywords:** BCLC stages, hepatocellular carcinoma, network meta-analysis

## Abstract

**Background:**

Currently, the Barcelona Clinic Liver Cancer staging (BCLC) system still remains controversies in the management of hepatocellular carcinoma. We are trying to determine the best therapeutic strategy for each BCLC stage through a network meta-analysis and provide a new treatment idea.

**Methods:**

We conducted a systematic literature search of the PubMed, EMBASE, and Cochrane Library databases and extracted data from randomized controlled trials (RCTs) that compared various strategies. Network meta-analyses were conducted in ADDIS by evaluating different overall survival of each stage. Cumulative probability was used to rank the included strategies. A node-splitting model assessed whether direct and indirect evidence on a specific node was in agreement.

**Results:**

Of the 24 included RCTs, 3667 patients were included. Based on the probability *P* values, the results showed that TACE plus surgical resection (SR) was the first choice for BCLC Stage A (*P* = .38 and *P* = .52 for 3- and 5-year OS, respectively). The application of SR was the best strategy for BCLC Stage B (*P* = .51 and *P* = .95 for 1- and 3-year OS, respectively). For Stage C, whole net connections could not be established in this research, but combined therapy seemed to produce better results based on 3 separated net connections (*P* = .92, *P* = .80, and *P* = .69 for 1-year OS).

**Conclusions:**

The updated therapy strategies discussed in this study are recommended. More importantly, we deemed that the recommended strategy for each patient may be subject to adjustment due to individual clinical factors. The applicable scope of each strategy should also be evaluated before application.

## Abstract 2

Supplemental Digital Content is available in the text

## 1 Introduction

Hepatocellular carcinoma (HCC), which is caused by viruses, cirrhosis, alcohol, and chemical toxins, is one of the most common malignancies worldwide and it is associated with high morbidity and mortality.^[1]^ Multiple local or systematic therapies have been employed for the treatment of HCC and surveillance programs have improved the early detection of HCC and decreased tumor-related mortality.^[2]^ However, most patients with HCC are diagnosed when in the intermediate to advanced stages, which is associated with a poor prognosis.^[3]^ Treatment options for HCC are limited and confusing. Therefore, it is important to establish a good HCC management system to provide the best therapy strategies for each patient.

The Barcelona Clinic Liver Cancer (BCLC) staging system, which establishes the prognosis and the best treatment strategy for patients in different stages, is widely used globally since it was first introduced.^[4]^ After 2 modifications,^[5,6]^ it became the standard specification for treatment of patients in different stages. The BCLC system is beneficial for HCC patients, and its obvious advantage is that it provides therapy options for each patient. Therefore, it is a complete management system and is strongly recommended. However, some scholars have suggested that the BCLC Therapeutic Flow-Chart is too conservative, and some have recommended the treatments should even be replaced.^[7]^ They have argued their viewpoints depending on randomized controlled trials (RCTs), cohort observation research, and meta-analysis. Until now, however, there has been no systematic comprehensive quantitative evidence to determine the best therapy strategy for each BCLC stage.

In recent years, many RCTs on strategies for HCC within or beyond the BCLC system recommendations have been published in different nations. Various treatment strategies showed different advantages and the controversy still remains. In this study, based on objective data, we performed a network meta-analysis with approaches that are currently regarded as the best tools for summarizing extant scientific evidence to determine the best therapy strategy for each BCLC stage. Most importantly, the objective of this study was to provide a new treatment idea that is beyond the specific therapeutic strategy itself.

## 2 Methods

### 2.1 Data sources and search strategy

This review was conducted using a predefined protocol and was in accordance with PRISMA and MOOSE guidelines.^[8,9]^ Global databases (PubMed, EMBASE, and Cochrane Central) were searched until August 1, 2016, without language or publication status restriction. The search started with the major search keys, namely “hepatocellular carcinoma (or HCC and liver cancer),” “overall survival,” and “randomized controlled trial (or RCT)” and then was expanded to relevant topics to avoid neglecting eligible studies. All abstracts available in English as well as non-English abstracts were reviewed, and the full text was consulted as necessary to clarify eligibility status. We limited attention to the various first-line or potential first-line therapy methods for HCC. Two independent investigators (Kun L and Yukun H) screened the titles and abstracts of all studies that were initially identified. Full texts were also retrieved independently from studies that satisfied all selection criteria. All retrieved articles with full texts (including controversial papers) were reserved for a final discussion for the included articles in the meta-analysis. The controversial papers were discussed by three investigators (Kun L, Yukun H, and Haitao W) and the final decision was made by the director (Tao G).

### 2.2 Study selection and eligibility criteria

**Figure 1:**
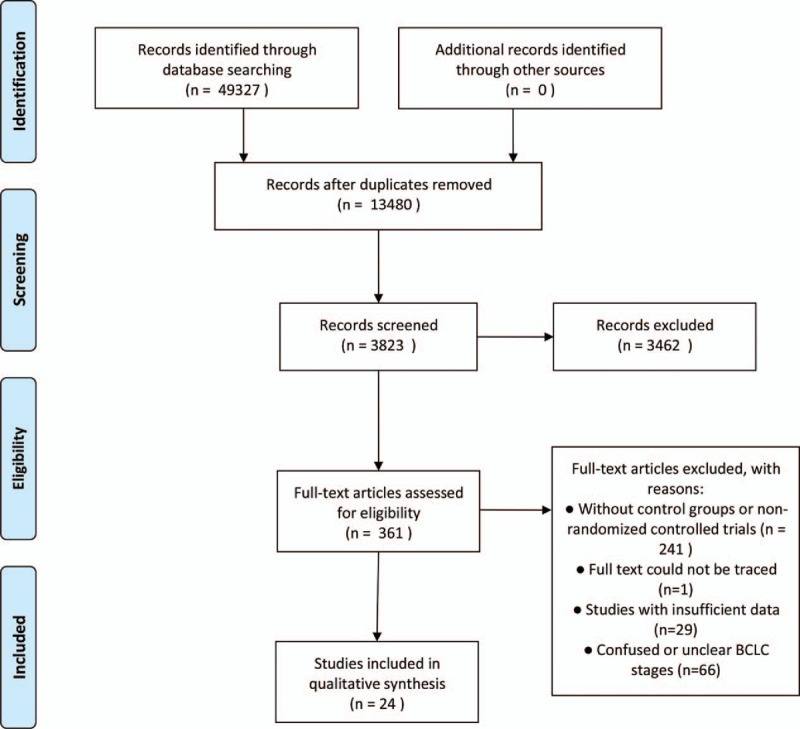
Flow diagram of the study selection procedures.

This research focused on different treatment regimens of HCC so that intervention studies were eligible if they were randomized clinical trials. Treatment methods were the only intervention considered for each study. In addition, due to the aim of this study, which was to determine the best treatment method for respective BCLC stages, RCTs of mixed or unclear BCLC stages were excluded. We paid attention to overall survival (OS) as the only parametric index to evaluate different therapies for respective BCLC stages so that an RCT that did not provide related data would also be excluded. Moreover, since the objective of this research was to evaluate the potential effects of different treatments for primary HCC, the trials on recurrent or metastatic HCC (from the colon, for instance) were not included. For the record, we only compared different therapy strategies and ignored the details of each strategy. The articles that compared different details of the same therapy (such as different drug doses or treatment cycles) were not included. Lastly, non-RCTs, pure cohort studies without control groups, description of animal models or experiments using cells, limitations to pure antiviral therapy, review articles and comment articles were excluded. We also applied a restriction to the length of follow-up, that is, at least a 1-year follow-up was required, and there was no restriction on the sample size in each group. The flow diagram of the process of selecting studies for this meta-analysis is presented in Fig. 1.

### 2.3 Data extraction

For the full-text articles that were retrieved, 2 investigators (Haitao W and Yukun H) independently reviewed and checked the included studies to assess the available data and randomization. A predesigned electronic data abstraction form was used to extract relevant general information (e.g., authors and year of publication) and parametric data (e.g., study arms and sample size of each group). The OS data of each eligible RCT study, including 1-year OS, 3-year OS, and 5-year OS for BCLC Stage 0 and Stage A; 1-year OS, 3-year OS for BCLC Stage B; 1-year OS for Stage C and Stage D, were extracted. Each included article was read in detail to screen out related information. For some RCTs, data were only extracted from subgroups or part of the study. The RCTs including different BCLC stages were recorded in different catalogs in the predesigned electronic database. Each study arm of the included trials was classified according to different treatment methods, ignoring the treatment details (drug dosage, repeated cycles, or treatment site).

### 2.4 Assessing the risk of bias

Two reviewers (Kun L and Haitao W) independently rated the quality of studies. Cochrane collaboration tool was used to assess the risk of bias.^[10]^ The controversial items were discussed with the director (Tao G), who made the final decision.

### 2.5 Statistical analysis

In this study, we paid close attention to the OS of different interventions for primary HCC with considering the BCLC stages. It was necessary to make comparisons among all therapy strategies via a comprehensive network meta-analysis based on the Bayesian theorem, which can be considered to be an extension of the traditional pair-wise meta-analysis as it incorporates both direct and indirect information through a common comparator to obtain estimates of the relative interventional effects on multiple intervention comparisons.^[11,12]^ Data on 1-, 3-, and 5-year OS in each study arm of the respective BCLC stages were recorded and collected for a pooled estimation based on network meta-analysis. We evaluated consistency by combining the quantitative estimates from direct and indirect comparisons according to the experimental design and primary outcomes of the included studies. Meanwhile, node-splitting analysis was also performed to show there was no statistical inconsistency when *P* was greater than .05. If there was no relevant inconsistency in the evidence, a consistency model was used to draw conclusions about the relative effect of the included interventions. An accumulated probability plot of *P*-value rankings showed the best therapeutic measures. For certain BCLC stages, if the included intervention connections could not be established as a whole net, the results were revealed as separated net connections or direct comparisons and were described together comprehensively.

RevMan5.3, provided by The Cochrane Library, was used for the description of risk of bias. The automated software Aggregate Data Drug Information System (ADDIS, version 1.16, GZ Groningen, Netherlands) was used for the network pooled estimation.

### 2.6 Ethical review

Ethical approval was not necessary, because this article is a meta-analysis and it does not involve the participation of ethics committee.

## 3 Results

### 3.1 Study characteristics and bias assessments

Through the literature search and selection based on the criteria above, we identified 49,327 relevant citations, and finally 24 RCTs^[13–36]^ were included in this meta-analysis (Fig. 1). The 24 RCTs reported BCLC stages of A–C (14 for Stage A, 3 for Stage B, 7 for Stage C), and RCTs with Stage 0 or Stage D did not exist (Table 1). All these RCTs reported results for 3667 unique patients (actual included sample size) with primary HCC, and the reports were published from 1996 to 2016. Eighteen RCTs were based in Asia, 5 were in Europe, and 1 was based in the USA. All included therapy strategies contained radiofrequency thermal ablation (RFA), percutaneous ethanol injection (PEI), percutaneous acetic acid injection (PAI), transcatheteral arterial chemoembolization (TACE), surgical resection (SR), percutaneous iodine-125 (^125^I), sorafenib, stereotactic body radiotherapy (SBRT), portal vein chemotherapy (PVC), cryotherapy (CR), or ginsenoside Rg3 (GRg3), and some combined methods (Table 1). All solitary methods and some combined strategies were treated as first-line or potential first-line treatments.

**Table 1:**
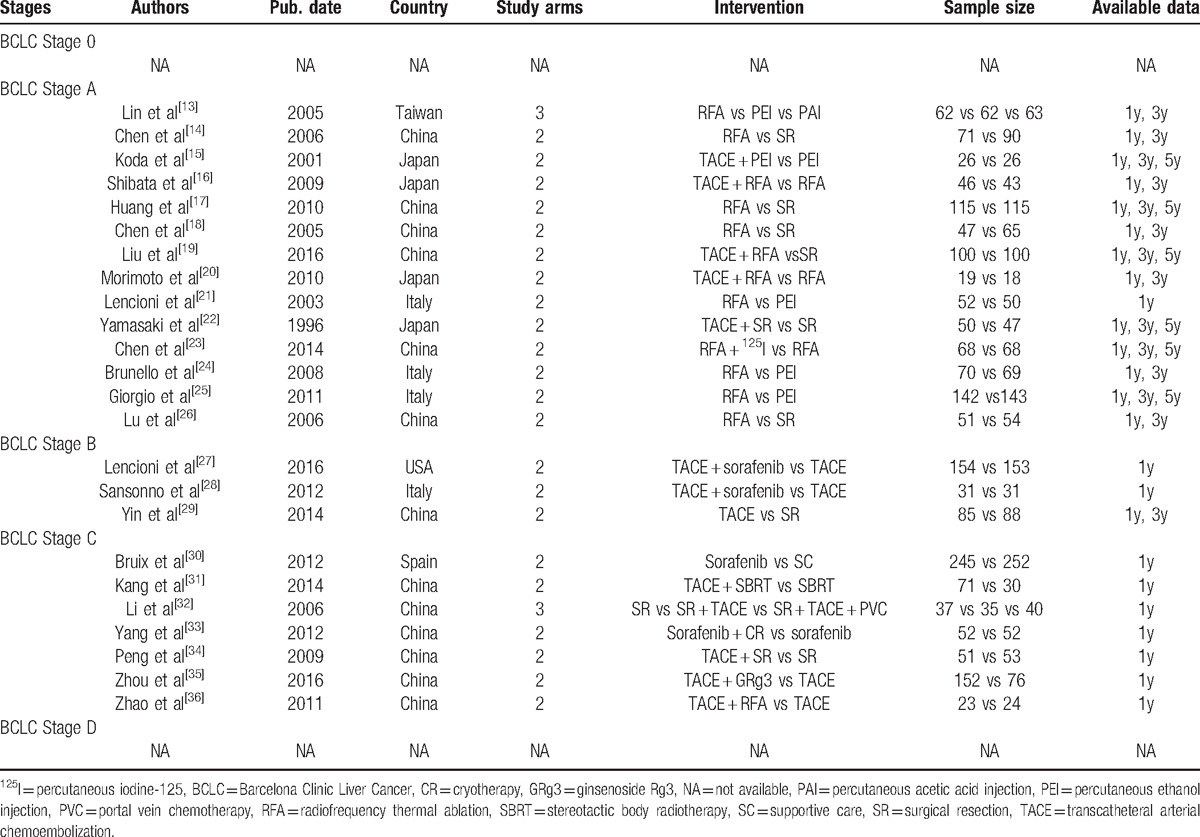
Characteristics of the included trials.

For assessments of bias, random sequence generation was clear in all included RCTs. For selection bias assessments, allocation concealment was detected in 9 of 24 RCTs, which may have revealed a high bias for BCLC Stage B. Only 7 RCTs clearly reported binding of participants and personnel. Meanwhile, a high bias risk for binding of outcome assessment may potentially exist in Catalog BCLC Stages C. On the other hand, low risk of attrition bias and reporting bias were demonstrated in each BCLC stage. Lastly, other bias was still unclear for most RCTs (Supplement Fig. S1).

### 3.2 Network meta-analysis of different overall survival for respective BCLC stages

For BCLC Stage 0 and D, no appropriate research was addressed for analyzing in this research due to our restrict standards.

**Figure 2:**
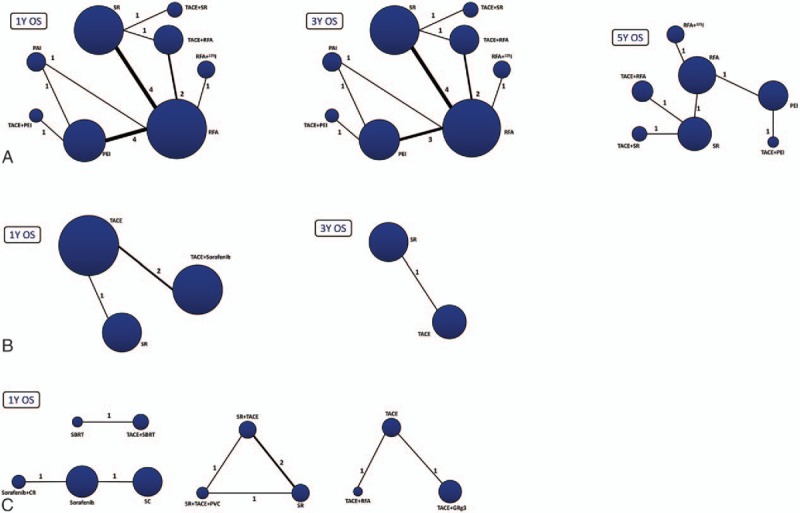
Network connections of included RCTs for (A) BCLC Stage A; (B) BCLC Stage B; and (C) BCLC Stage C. The numbers on the line indicate the quality of studies compared with every pair of treatments, which were also represented by the width of the lines. Also, the sizes of the areas of the circles stand for the respective sample sizes.

**Figure 3:**
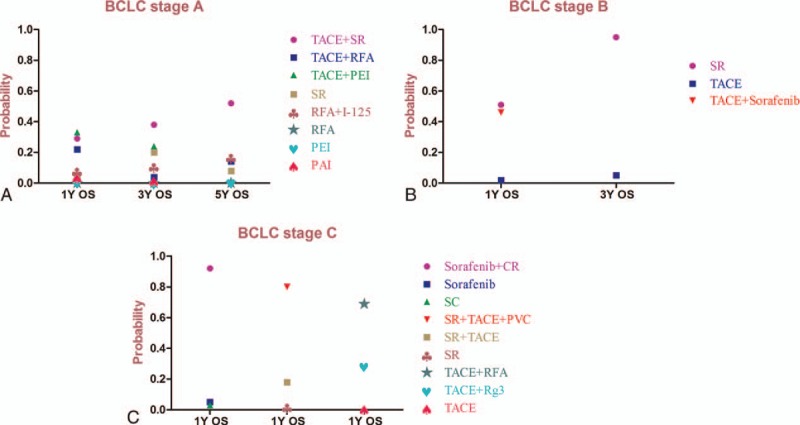
Probability of different therapy strategies as measured by the included outcomes for BCLC (A) Stage A; (B) Stage B; and (C) Stage C.

For patients with BCLC Stage A, 14 RCTs reported 1-, 3-, and 5-year OS (14, 13, and 6 reported 1-, 3-, and 5-year OS, respectively). We conducted a network meta-analysis for different OS by establishing 3 network connections (Fig. 2 A). It was shown that TACE plus PEI may be the most effective therapy to improve 1-year OS for patients with BCLC Stage A (*P* = .29). However, for longer OS comparisons, that is, 3- and 5-year OS, TACE plus SR became the best method (*P* = .38 and *P* = .52, respectively; Supplement Table S1 and Fig. 3A).

There were 3 RCTs addressed for comparisons of 1- and 3-year OS of BCLC Stage B. The related network connections are presented in Fig. 2B. The results of the meta-analysis revealed that SR was the most effective therapy for patients with BCLC Stage B to improve the 1- and 3-year OS (Supplement Table S1) and could potentially be the best strategy (*P* = .51 and *P* = .95, respectively; Fig. 3B).

For BCLC Stage C patients, we only made a comparison of 1-year OS and the whole network connection could not be established. The total 7 RCTs were divided into 1 direct comparison and 3 separated net connections for indirect comparisons (Fig. 2C). The direct comparison and related 3 meta-analyses (Supplement Table S1) revealed that sorafenib plus CR, SR plus TACE plus PVC, and TACE plus RFA produced better results than other treatment strategies (Probability *P* = .92, *P* = .80 and *P* = .69, respectively) (Fig. 3C) but the related best strategy was unclear.

### 3.3 Node-splitting analysis of inconsistency

Node-splitting models were conducted to assess inconsistency by testing the differences between the direct and indirect effects. This analysis assessed whether direct and indirect evidence for a specific node (the split node) were in agreement. After constructing the node-splitting models for only BCLC Stage A, which could potentially exist as an inconsistency, we found that no significant inconsistency existed in this research (*P* > .05, for all) and the results of the consistency model were reliable (Supplement Table S2).

## 4 Discussion

The BCLC staging system has come to be widely accepted in clinical practice and is also being used for many clinical trials of new drugs to treat HCC. On the other hand, although the BCLC staging system is innovative and includes several aspects of HCC biology and underlying liver disease, its general application remains a matter of ongoing discussion, especially in the case of potentially resectable lesions.^[37]^ Furthermore, SR for early BCLC stage patients has been treated more invasively than local therapy and significant advantages for each patient were not revealed.^[38]^ Meanwhile, for advanced stage patients, SR and combined therapy were also advocated.^[39,40]^ The question is, although many scholars and experts have debated the specific treatment methods for each BCLC stage, their theories were only based on some published RCTs, observation studies, authoritative opinions and personal experiences. In other words, so far, there has been no quantitative statistical evidence and systematic objective judgment to provide references for this argument.

To achieve accuracy and reduce information bias in this review, we only included the RCTs that had been published. After comparisons of various therapy strategies for each specific BCLC stage based on network meta-analysis, the results revealed the best strategies for each stage except for BCLC Stage 0 and Stage D. TACE plus SR was demonstrated to be superior to other therapy strategies for patients with Stage A. And SR was the best strategy to treat BCLC Stage B patients. Finally, for Stage C patients, no best therapy strategy was determined. However, sorafenib plus CR, SR plus TACE plus PVC, and TACE plus RFA were superior to other reported strategies (Fig. 3).

Based on the objective results, for BCLC Stage A, SR, liver transplantation (LT), and RFA were replaced by TACE plus SR. In addition, SR revealed obvious advantages for BCLC Stage B. These results may indicate some potential facts. First of all, a solitary SR strategy may be not appropriate for early stage patients. On the contrary, a SR strategy should expand the scope of its application to Stage B and C, as mentioned above. Secondly, combined with TACE, TACE plus SR was more suitable for Stage A but not in Stage B. This result may occur because patients who underwent SR with Stage B (large or multiple lesions) may have worse hepatic functional reservation compared with Stages A. Meanwhile, for early HCC lesions (Stage A), TACE combined with minimally invasive therapy, PEI, was better than combined with invasive therapy, SR, for 1-year OS. But for long-term OS (3- and 5-year), TACE plus SR revealed its advantages and became the first choice. This may clarify that minimally-invasive therapy was the best choice for early survival rate, but SR seemed to be a more thorough treatment, combined with TACE. As tumor stage progresses, SR becomes increasingly more effective than others combined with TACE (in the case of good hepatic functional reserve). When tumor stages progress further, SR could bring more impact on hepatic functional reservation and, combined with TACE, may not be suitable. Lastly, for Stage C patients, we did not address the best strategy because the whole network connection could not be established. However, we addressed 3 relative superior methods (sorafenib plus CR, SR plus TACE plus PVC, and TACE plus RFA). Based on the results of Stage C, we concluded that pure solitary treatment strategies were inappropriate for patients with Stage C, although the best strategy was not identified based on current objective data. Moreover, some scholars indeed believed that combination or systemic therapies should be applied for the patients with advanced HCC.^[41]^ This may be an indication that combined or systematic therapy should be recommended for this stage.

For the record, we must admit that there were several inevitable limitations that existed in this research. First, although we extended our search scope, the included RCTs were still insufficient (especially for Stage 0 and D). Moreover, many first line or potential first-line strategies (in our experience, such as SR or LT) could not be included due to the exclusion of many articles of mixed or unclear BCLC stages. So the best strategy for some certain stages may need to be updated in the future (such as Stage 0 and A). Furthermore, due to the own defects of BCLC system,^[42,43]^ the uncertain boundaries of some stages also leads to controversy.^[44–46]^ Last, due to the limitations of the literature retrieval strategies and inclusion criteria of this investigation, we may have overlooked some defects in study designs, potential bias, and results interpretations. To various degrees, all of these aforementioned confounding factors might have contributed to our final conclusions.

**Figure 4:**
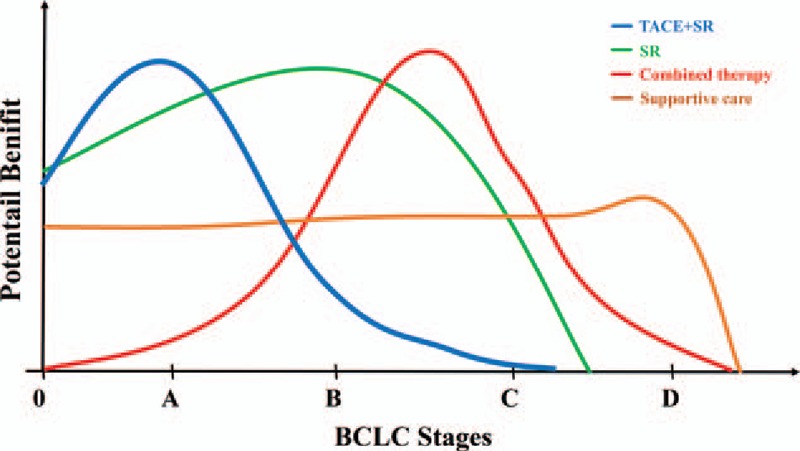
Potential benefit curves for each strategy for different tumor stages based on the objective results.

We have interpreted the objective results of this study (Table 2) and also pointed out deficiencies. It needs to be acknowledged that the RCTs included the highest level of evidence, but they were not easy to perform in some fields because of ethical and practical conditions. Still, we may present some new ideas for HCC therapy based on the objective results of this study. While debating the best specific strategy for each BCLC stage, we may have neglected that each strategy was only suitable for some conditions, which may not apply to everyone, even in same stage. For instance, patients with portal vein tumor thrombus (PVTT) were recommended to undergo TACE according to the BCLC Therapeutic Flow-Chart. However, in this research, we could conclude that adding RFA (recommended for Stage A before) to establish combined therapy for Stage C patients was better than TACE (Fig. 3C). Combining these objective data and our hypotheses, we suggest that each therapy could bring benefits at each stage, but the potential benefits for each stage are graded: TACE plus SR may bring the best effects for Stage A. However, when tumors progress to the next stage, TACE should be limited and pure SR may be the best choice. In the next stage, due to complicated conditions, combined therapy revealed advantages. When the end stage was considered, only supportive care (SC) revealed effects (Fig. 4). These semi-quantitative benefit curves were described based on our objective results and hypotheses. We understood that debate could not be completely eliminated in this research and empiricism may also remind us that there may be a better treatment for each stage. However, our results were derived from the existing objective evidence-based medical information. They may not completely accurate for managing every patient but offered new ideas for managing HCC. We believe they will keep changing as time goes on and treatment technology improves. Most important, in our opinion, HCC management was not the specific best strategy for each individual, but it aided in the understanding of the approximate scope of applications and choosing the most suitable treatment for a specific patient.

**Table 2:**
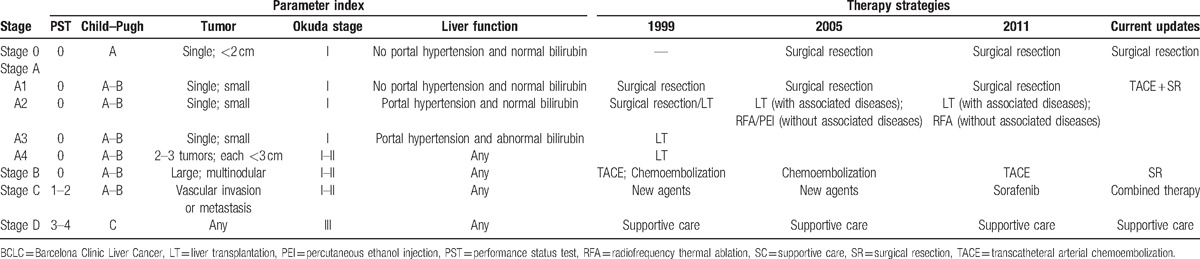
The BCLC stage system and the updated strategies in this study.

Despite the existence of several limitations, we updated the HCC strategies for the BCLC staging system (Table 2) based on objective data from current RCTs. More importantly, we provided a new paradigm as a reference scope to consider the relevant potential benefit for each patient.

## Supplementary Material

Supplemental Digital Content
