# Supplementary material for: New idea for treatment strategies for Barcelona Clinic Liver Cancer stages based on a network meta-analysis
Source: Medicine (Baltimore). 2017 May 19;96(20):e6950. doi: 10.1097/MD.0000000000006950 (PMC5440153; doi:10.1097/MD.0000000000006950)
Supplement: Supplemental Digital Content [file medi-96-e6950-s001.pdf]

A

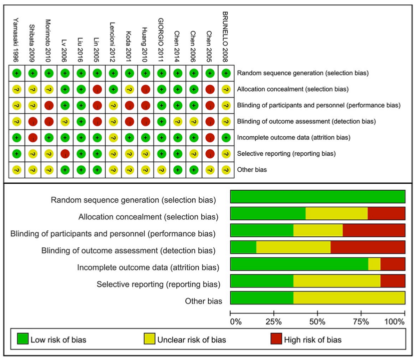
Yamasaki 1996
Shibata 2009
Morimoto 2010
Lv 2006
Liu 2016
Lin 2005
Lencioni 2012
Koda 2001
Huang 2010
GIORGIO 2011
Chen 2014
Chen 2006
Chen 2005
BRUNELLO 2008
Random sequence generation (selection bias)
Allocation concealment (selection bias)
Blinding of participants and personnel (performance bias)
Blinding of outcome assessment (detection bias)
Incomplete outcome data (attrition bias)
Selective reporting (reporting bias)
Other bias
0%
25%
50%
75%
100%
Low risk of bias
Unclear risk of bias
High risk of bias

B

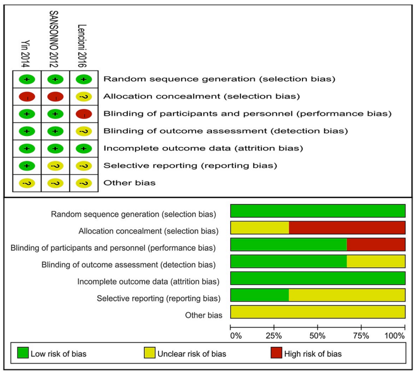
Yin 2014
SANSONNO 2012
Lencioni 2016
Random sequence generation (selection bias)
Allocation concealment (selection bias)
Blinding of participants and personnel (performance bias)
Blinding of outcome assessment (detection bias)
Incomplete outcome data (attrition bias)
Selective reporting (reporting bias)
Other bias
0%
25%
50%
75%
100%
Low risk of bias
Unclear risk of bias
High risk of bias

C

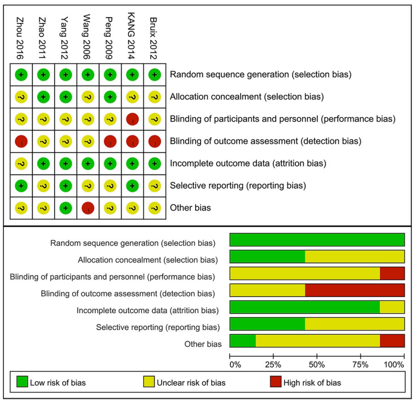
Zhou 2016
Zhao 2011
Yang 2012
Wang 2006
Peng 2009
KANG 2014
Bruix 2012
Random sequence generation (selection bias)
Allocation concealment (selection bias)
Blinding of participants and personnel (performance bias)
Blinding of outcome assessment (detection bias)
Incomplete outcome data (attrition bias)
Selective reporting (reporting bias)
Other bias
0%
25%
50%
75%
100%
Low risk of bias
Unclear risk of bias
High risk of bias

Supplement Table S1. The Network Meta-analysis Results of Therapy Strategies for Each BCLC stage.

Items	Comparisons

BCLC Stage A. 1-year overall survival

PAI	1.02 (0.10, 9.12)	0.83 (0.09, 8.58)	0.85 (0.02, 37.45)	1.63 (0.14, 24.54)	2.76 (0.04, 283.02)	2.50 (0.11, 118.04)	2.84 (0.06, 189.09)
0.98 (0.11, 10.43)	PEI	0.83 (0.24, 3.92)	0.83 (0.03, 22.99)	1.63 (0.28, 13.99)	2.79 (0.07, 168.84)	2.55 (0.18, 74.19)	2.83 (0.09, 129.12)
1.20 (0.12, 11.25)	1.20 (0.25, 4.21)	RFA	0.99 (0.05, 19.34)	1.95 (0.55, 7.53)	3.34 (0.07, 229.48)	3.08 (0.29, 57.42)	3.35 (0.13, 104.56)
1.18 (0.03, 46.55)	1.20 (0.04, 29.19)	1.01 (0.05, 18.45)	RFA+125I	1.99 (0.09, 50.94)	3.30 (0.03, 515.41)	3.13 (0.07, 182.90)	3.32 (0.05, 300.25)
0.61 (0.04, 7.06)	0.62 (0.07, 3.58)	0.51 (0.13, 1.81)	0.50 (0.02, 11.46)	SR	1.69 (0.03, 144.20)	1.57 (0.16, 23.13)	1.74 (0.09, 40.54)
0.36 (0.00, 24.13)	0.36 (0.01, 13.78)	0.30 (0.00, 14.95)	0.30 (0.00, 37.89)	0.59 (0.01, 39.21)	TACE+PEI	0.96 (0.01, 131.72)	0.99 (0.00, 194.77)
0.40 (0.01, 9.10)	0.39 (0.01, 5.54)	0.32 (0.02, 3.49)	0.32 (0.01, 13.74)	0.64 (0.04, 6.14)	1.04 (0.01, 130.45)	TACE+RFA	1.09 (0.02, 48.57)
0.35 (0.01, 18.17)	0.35 (0.01, 11.44)	0.30 (0.01, 7.68)	0.30 (0.00, 21.99)	0.57 (0.02, 11.20)	1.01 (0.01, 210.93)	0.92 (0.02, 56.89)	TACE+SR

BCLC Stage A. 3-year overall survival

PAI	0.94 (0.23, 3.77)	1.21 (0.29, 5.08)	1.21 (0.09, 16.23)	2.92 (0.61, 14.98)	2.11 (0.21, 22.75)	1.66 (0.31, 11.28)	3.13 (0.33, 33.90)
1.06 (0.27, 4.31)	PEI	1.30 (0.52, 3.19)	1.28 (0.11, 14.25)	3.10 (1.01, 10.29)	2.23 (0.35, 14.83)	1.77 (0.48, 8.23)	3.39 (0.44, 28.23)
0.82 (0.20, 3.41)	0.77 (0.31, 1.94)	RFA	0.98 (0.10, 9.04)	2.41 (1.19, 5.23)	1.73 (0.21, 14.32)	1.38 (0.52, 4.54)	2.59 (0.41, 17.35)
0.82 (0.06, 11.52)	0.78 (0.07, 9.12)	1.02 (0.11, 9.82)	RFA+125I	2.42 (0.24, 25.79)	1.74 (0.09, 39.14)	1.43 (0.12, 17.97)	2.69 (0.14, 50.66)
0.34 (0.07, 1.65)	0.32 (0.10, 0.99)	0.42 (0.19, 0.84)	0.41 (0.04, 4.24)	SR	0.72 (0.08, 6.62)	0.57 (0.20, 1.92)	1.08 (0.19, 6.22)
0.47 (0.04, 4.88)	0.45 (0.07, 2.87)	0.58 (0.07, 4.76)	0.57 (0.03, 11.69)	1.38 (0.15, 12.98)	TACE+PEI	0.79 (0.08, 9.00)	1.49 (0.09, 24.25)
0.60 (0.09, 3.24)	0.57 (0.12, 2.08)	0.72 (0.22, 1.92)	0.70 (0.06, 8.22)	1.76 (0.52, 4.89)	1.26 (0.11, 11.95)	TACE+RFA	1.90 (0.23, 14.14)
0.32 (0.03, 3.06)	0.29 (0.04, 2.29)	0.39 (0.06, 2.41)	0.37 (0.02, 6.96)	0.93 (0.16, 5.13)	0.67 (0.04, 10.55)	0.53 (0.07, 4.41)	TACE+SR

BCLC Stage A. 5-year overall survival

PEI	1.08 (0.31, 3.87)	2.14 (0.34, 14.14)	2.84 (0.46, 16.92)	1.19 (0.24, 5.77)	2.54 (0.26, 21.25)	4.15 (0.43, 39.29)
0.92 (0.26, 3.25)	RFA	2.00 (0.51, 7.84)	2.61 (0.71, 9.28)	1.07 (0.15, 8.71)	2.32 (0.36, 13.43)	3.82 (0.56, 24.42)
0.47 (0.07, 2.92)	0.50 (0.13, 1.97)	RFA+125I	1.31 (0.18, 8.62)	0.55 (0.04, 6.51)	1.15 (0.11, 11.18)	1.90 (0.17, 20.22)
0.35 (0.06, 2.17)	0.38 (0.11, 1.42)	0.77 (0.12, 5.48)	SR	0.42 (0.04, 4.71)	0.88 (0.25, 3.12)	1.48 (0.36, 5.82)
0.84 (0.17, 4.10)	0.93 (0.11, 6.86)	1.82 (0.15, 22.27)	2.39 (0.21, 28.36)	TACE+PEI	2.15 (0.13, 33.49)	3.58 (0.22, 58.18)
0.39 (0.05, 3.85)	0.43 (0.07, 2.82)	0.87 (0.09, 9.16)	1.14 (0.32, 4.02)	0.47 (0.03, 7.66)	TACE+RFA	1.66 (0.25, 11.37)
0.24 (0.03, 2.35)	0.26 (0.04, 1.80)	0.53 (0.05, 5.92)	0.67 (0.17, 2.80)	0.28 (0.02, 4.59)	0.60 (0.09, 4.02)	TACE+SR

BCLC Stage B. 1-year overall survival

SR	0.32 (0.02, 6.15)	0.92 (0.03, 49.27)
3.15 (0.16, 65.41)	TACE	2.97 (0.40, 40.00)
1.09 (0.02, 35.81)	0.34 (0.03, 2.53)	TACE+Sorafenib

BCLC Stage B. 3-year overall survival

SR	0.18 (0.02, 1.53)
5.55 (0.65, 49.58)	TACE

BCLC Stage C. 1-year overall survival (Sorafenib vs. SC vs. Sorafenib+CR)

SC	1.81 (0.47, 6.84)	5.73 (0.69, 45.35)
0.55 (0.15, 2.15)	Sorafenib	3.11 (0.67, 15.36)
0.17 (0.02, 1.45)	0.32 (0.07, 1.49)	Sorafenib+CR

BCLC Stage C. 1-year overall survival (SR+TACE vs. SR vs. SR+TACE+PVC)

SR	1.86 (0.72, 5.05)	3.12 (0.84, 12.32)
0.54 (0.20, 1.39)	SR+TACE	1.68 (0.43, 6.70)
0.32 (0.08, 1.19)	0.59 (0.15, 2.34)	SR+TACE+PVC

BCLC Stage C. 1-year overall survival (TACE+RFA vs. TACE vs. TACE+Rg3)

TACE	4.06 (0.58, 29.00)	2.19 (0.40, 12.60)

0.25 (0.03, 1.72)	TACE+RFA	0.55 (0.04, 6.95)
0.46 (0.08, 2.50)	1.82 (0.14, 24.57)	TACE+GRg3

Supplement Table S2. Results of Node-splitting Models for the Test of Difference Between Direct and Indirect Effect in the Analysis of Primary Outcomes.

Items	Comparison	Direct Effect	Indirect Effect	Overall	*P* value
**BCLC Stage A. 1-year**	**overall survival**				
	**RFA, SR**	0.49 (-0.84, 1.80)	17.61 (1.44, 61.34)	0.68 (-0.59, 2.02)	0.16
	**RFA, TACE+RFA**	22.49 (1.76, 56.91)	0.19 (-2.99, 3.31)	1.13 (-1.25, 4.05)	0.16
	**SR, TACE+RFA**	-0.34 (-3.26, 2.41)	19.06 (1.08, 90.92)	0.45 (-1.81, 3.14)	0.11
**BCLC Stage A. 3-year**	**overall survival**				
	**RFA, SR**	0.83 (-0.00, 1.71)	1.28 (-0.77, 3.59)	0.88 (0.18, 1.65)	0.65
	**RFA, TACE+RFA**	0.54 (-0.84, 2.11)	0.06 (-1.82, 2.02)	0.32 (-0.65, 1.51)	0.64
	**SR, TACE+RFA**	-0.76 (-2.42, 0.98)	-0.31 (-1.92, 1.54)	-0.56 (-1.59, 0.65)	0.65
